# Adherence to Dietary Recommendation and Its Associated Factors among People with Type 2 Diabetes: A Cross-Sectional Study in Nepal

**DOI:** 10.1155/2022/6136059

**Published:** 2022-10-21

**Authors:** Jijeebisha Baral, Khem Bahadur Karki, Pratibha Thapa, Ashish Timalsina, Rama Bhandari, Rabindra Bhandari, Bijaya Kharel, Nabin Adhikari

**Affiliations:** ^1^Central Department of Public Health, Institute of Medicine, Tribhuvan University, Kathmandu, Nepal; ^2^Department of Community Medicine, Institute of Medicine, Tribhuvan University, Kathmandu, Nepal; ^3^Department of ENT, Tribhuvan University Teaching Hospital, Kathmandu, Nepal; ^4^Dhulikhel Hospital, Research and Development Department, Dhulikhel, Kavrepalanchok, Nepal

## Abstract

**Introduction:**

Intensive lifestyle modification including a healthy diet changes the diagnostic status of patient from prediabetes to nondiabetes. In type 2 diabetes, improper eating habits increase insulin resistance. This study is aimed at assessing adherence to the dietary recommendation and its associated factors among people with type 2 diabetes.

**Methods:**

A cross-sectional descriptive study was conducted among systematically sampled type 2 diabetic patients using interview on Gandaki Medical College Teaching Hospital and Diabetes, Thyroid, and Endocrinology Care Center, Pokhara. The Perceived Dietary Adherence Questionnaire was used to assess dietary adherence. Data was entered in EpiData version 3.1 and analyzed on SPSS version 20. Logistic regression with adjusted odds ratio and the corresponding 95% confidence intervals were used to find out significance of association.

**Results:**

Among 204 participants, only 15.7% of the participants had good dietary adherence. The mean age and standard deviation were 53.03 ± 11.90 years. Factors such as participants living in single family (AOR 2.7, 95% CI 1.0-7.4), participants who could afford recommended diet (AOR 2.9, 95% CI 1.0-8.3), participants having self-control on food (AOR 4.1, 95% CI 1.2-14.1), participants who were engaged in moderate to heavy physical activities (AOR 3.3, 95% CI 1.2-9.2), and participants who had adherence to medication (AOR 3.5, 95% CI 1.2-10.1) were significantly associated with adherence to dietary recommendation.

**Conclusions:**

Adherence to dietary recommendation among people with type 2 diabetes was low. Factors such as family type, affordability of recommended diet, self-control on food, physical activity, and medication adherence were significantly associated with adherence to dietary recommendations among people with type 2 diabetes. These factors should be considered by nutrition counselors and clinical decision-makers in patient counseling regarding dietary adherence.

## 1. Introduction

Diabetes is a chronic, metabolic disease characterized by elevated levels of blood glucose which over time causes major harm to the heart, blood vessels, eyes, kidneys, and nerves [1]. The risk of developing type 2 diabetes increases with age, obesity, improper diet, and lack of physical activity [2]. Adherence to dietary recommendations varied widely across countries ranging from 29.9% to 67.4% [3]. About 11 million deaths and 255 million DALYs were attributable to dietary risk factors, and among them, the impact of diet on mortality in type 2 diabetes is 338,714 deaths and 24 million DALYs [4]. In Nepal, diabetes is among the top ten causes of years lived with disability (YLDs) and death in 2017 [5]. There is 2.8% years of life loss in total due to noncommunicable diseases contributed to diabetes mellitus in Nepal in 2009 [6].

Diet is one of the essential treatment components and can lower glycated hemoglobin (HbA1c) levels by 1% to 2% [7]. The intensive lifestyle intervention including dietary adherence could reduce the incidence of type 2 diabetes by 58% [8]. A low-carbohydrate diet, a diet with low glycemic index, high-fiber diet, fruits and vegetables, and omega 3 fatty acid-containing food are effective in lowering blood glucose parameters in type 2 diabetes while saturated fatty acid and trans-fat decrease insulin sensitivity [9]. Appropriate dietary practices for type 2 diabetes patients include the intake of less fat, more fiber, less sodium, and more foods that have health-promoting properties such as fish, soy products, fruits, and vegetable [4]. The American Diabetes Association recommends that the carbohydrate intake should emphasize nutrient-dense carbohydrate sources that are high in fiber, including vegetables, fruits, legumes, and whole grains, as well as dairy products, advised to avoid sugar-sweetened beverages (including fruit juices), limiting the amount of dietary saturated and trans-fat intake, and include fat-free dairy product fish, poultry, beans, nuts, and vegetable oils [10].

Despite understanding the importance of dietary control and physical activity in the management of diabetes, adherence to practices has been poor [11]. Diet quality and quantity over the longer term are relevant to the prevention and management of diabetes and its complications [12]. The studies on dietary adherence in Nepal are limited. This study will facilitate the context-specific understanding of the dietary adherence of diabetic patients which helps to identify the appropriate adherence solutions. This study will also assist the clinicians and managers for the management and control of type 2 diabetes from dietary perspective in Nepal.

## 2. Methods

### 2.1. Study Design and Study Sites

We conducted an institution-based cross-sectional study from 27 January 2020 to 12 March 2020 at the Gandaki Medical College Teaching Hospital and Diabetes, Thyroid, and Endocrinology Care Center of Pokhara Metropolitan City. The Gandaki Medical College Teaching Hospital is located in Prithvi Chowk, Ward no. 9, Pokhara. It is a tertiary health center with 450 bed capacity. The average flow of diabetes cases in the outpatient department was about 450 per month. The Diabetes, Thyroid, and Endocrinology Care Center is located in New Road, Pokhara. It is an outpatient-based health facility specialized for diabetes-, thyroid-, and endocrine-related cases. The average flow of diabetes cases was about 500 per month. These institutions were chosen based on the similarity of dietary recommendation they provide, with the tools that we have used in our study.

### 2.2. Study Population

The study population was type 2 diabetes visiting the outpatient department of the two selected health institutions (Gandaki Medical College Teaching Hospital and Diabetes, Thyroid, and Endocrinology Care Center).

### 2.3. Inclusion Criteria

People diagnosed with type 2 diabetes visiting the outpatient department of Gandaki Medical College Teaching Hospital and Diabetes, Thyroid, and Endocrinology Care Center were included in this study.

### 2.4. Exclusion Criteria

Newly diagnosed type 2 diabetes of less than one month, pregnant and lactating women, and those requiring constant medical support and monitoring were planned to be excluded from this study. During the data collection, none of the cases met these criteria. So, none of the participants were excluded from the study.

### 2.5. Sample Size and Sampling Technique

The sample size was determined using single population proportion formula. Considering the prevalence of dietary adherence 14.29% [13] at a 95% level of significance, 5% margin of error, and after adding 8% of calculated sample size for possible nonresponse, the final sample size was 204 for the study.

We used a systematic random sampling method to select the study participants. The average visit of type 2 diabetes per day was calculated from the previous hospital records. Then, the total participants within a month were estimated. The total participants were divided by the sample size to get the sampling interval which was 3. Based on the systematic random sampling, we selected every first and third participant for this study.

### 2.6. Study Variables

#### 2.6.1. Dependent Variables

The Perceived Dietary Adherence Questionnaire (PDAQ) tool was used for measuring the dietary adherence in this study. It is a nine-item questionnaire which was developed by Asaad et al. in 2015 [13]. The responses are based on a seven-point Likert scale to recall the food consumed in the last 7 days. Higher scores reflect higher adherence except for items 4 and 9, which reflect unhealthy choices (i.e., foods high in sugar or fat). For these items, higher scores reflect lower adherence; therefore, for computing a total PDAQ score, the scores for these items were recoded. Patients were classified as having good dietary adherence if they eat a healthy diet for at least four days in the week.

#### 2.6.2. Independent Variable

Independent variables were selected based on the previously published studies. Independent variables were broadly classified into sociodemographic factors, disease-related factors, individual factors, anthropometry, and medical adherence-related factors. Sociodemographic factors included age of the participant, sex, ethnicity, education, residence, marital status, religion, occupation, family type, and family size. Disease-related factors included duration of diagnosis, type of treatment, comorbidity, and family history of diabetes. Individual factors included affordability to a healthy diet, sugar-sweetened beverage preference, and self-control over food [14, 15].

Anthropometry measurement included the following: (1) body mass index (the cutoff for measurement of body mass index (BMI) was based on WHO classification): BMI is classified as normal if it lies between 18.5 and 24.9 kg/m^2^. It is considered as overweight if it lies between 25 and 29.9 kg/m^2^. BMI on obese class I is between 30 and 34.9 kg/m^2^. Obese class II BMI is between 35 and 39.9 kg/m^2^. Similarly, BMI on obese class III is greater than or equal to 40 kg/m^2^; (2) waist circumference (the cutoff for measurement of waist circumference for male and female was based on the American Diabetes Association 2007): waist circumference of greater than 85 cm in male and greater than 80 cm in female is considered abnormal; and (3) waist-hip ratio (the cutoff for measurement of waist-hip ratio for male and female is based on the American Diabetes Association 2007): a waist-hip ratio of greater than 0.90 is considered abnormal for male. Similarly, a waist-hip ratio of greater than 0.80 is considered abnormal for female.

The Diabetes Medication Adherence Scale (DMAS) was used as the tool for assessing the adherence to antidiabetic medication consisting of seven questions of response of “Yes” or “No.” For each response, “Yes” was rated as zero while one rating was done for response “No.” The value of seven was considered as adherence, and the value of less than seven was considered as nonadherence [16].

#### 2.6.3. Data Collection Tools and Technique

We conducted a face-to-face interview with participants using a structured questionnaire. We also used a stadiometer to measure the height and a weighing machine to measure the weight of participants. A pilot test was done in 10% of the nonsampled hospital, i.e., TUTH Hospital of Kathmandu, which was not included in the final survey. Necessary modification on tools was done based on feedbacks received from the pretesting. The questionnaire was evaluated for reliability with a Cronbach's value 0.718 which was found to be adequate. The questionnaire was composed of two parts. The first parts consist of structured questionnaire regarding sociodemographic factor, disease-related factor, individual factor, anthropometry, and medication adherence. In the second part, we assessed the dietary adherence measurement. This tool was validated with repeated 24 h dietary recall of type 2 diabetic patients. The intraclass correlation of this tool (0.78) indicates good reliability.

JB and two other trained enumerators were involved in data collection from 27 January to 12 March 2020. The average time for an interview was 25 minutes.

### 2.7. Statistical Analysis

The collected data were systematically coded and entered into EpiData version 3.1. The entered data were exported to IBM SPSS Version 20 where consistency was checked as well as cleaning and editing of data were done. Required analysis was performed in IBM SPSS version 20. Descriptive analyses (frequency and percentage) were used to report the dependent and independent variables. Frequency tables were used for categorical variables, while mean and standard deviation (SD) were calculated for continuous variables. Univariate and multivariate analyses were done. Those variables which were significantly associated in the univariate analysis at 95% level of confidence, *p* value less than 0.1, were included in the multivariate model. We applied multivariate logistic regression analysis adjusting for covariates such as education, occupation, type of families, diet preference, affordability of healthy diet, self-control on food, physical activity, and medical adherence to identify the factors associated with dietary adherence. The unadjusted and adjusted odds ratios with 95% confidence intervals were reported. *p* value less than 0.05 was considered statistically significant.

### 2.8. Ethical Considerations

Ethical approval for the study was obtained from the Nepal Health Research Council (Ref. 1839) and Institutional Review Board of Institute of Medicine (Ref. 336). Permission was taken from the selected hospitals. Permission was taken for using Diabetes Medication Adherence Scale and Perceived Dietary Adherence Questionnaire tools. The objective of the study was explained to the participants, and written consent was taken from the participants before data collection. The right of the participants who do not want to participate in the study was respected.

## 3. Results

### 3.1. Sociodemographic Characteristics

Table 1 shows the distribution of participants by their sociodemographic characteristics. Among the total 204 participants, the majority of them were female (56.9%). The participants within the age group of 45-64 years were the highest (61.2%) while the age 65 years and above of them were the lowest (18.7%). More than half (53.4%) were of Brahmin/Chhetri caste group. Regarding the education level, 32.8% were illiterate. Almost 9 out of 10 participants (88.7%) were married. In our study, the urban residents were higher than the rural ones (61.8% and 38.2%, respectively). Majority of participants followed Hindu (81.4%) religion. More than half of the participants (56.86%) were involved in non-income-generating work while more than half of them lived in a nuclear family (55.4%).

### 3.2. Distribution of Participants by Disease-Related, Dietary, Medication Adherence, and Anthropometric-Related Characteristics

Table 2 shows the distribution of participants by disease-related, dietary, medication adherence, and anthropometric-related characteristics. The participants having the disease for less than and equal to five years were 57.4%. The majority of the participants (89.2%) were on oral hypoglycemic drug while 10.8% were taking both oral and insulin. More than half of the participants (54.9%) had comorbidity while the majority (74.5%) had no family history of diabetes. More than half (52.5%) had no preference to sugar-sweetened beverage. About half of the participants (51.5%) could afford the prescribed diet. Majority of the participants (65.2%) reported self-control on food. More than two-fifths of the participants (44.6%) had BMI between 25 and 29.9 kg/m^2^. The majority of male participants (63.64%) had abnormal waist circumference of >85 cm. A majority (66.38%) of female participants had abnormal waist circumference of >80 cm. In the waist-hip ratio, most of the participants (90.91%) had ratio greater than 0.90 among males. In females, 90.52% had greater than 0.80 waist-hip ratio. Nearly half (48%) of the participants were nonadherent to medication. Only 15.7% of the participants had good adherence to diet.

### 3.3. Factors Associated with Dietary Adherence

Table 3 shows the factors associated with dietary adherence. From the regression analysis, we found that the participants living in single families were almost 3 times more likely to have adherence to diet than those on joint families (AOR 2.7, 95% CI 1.0-7.4). The participants who could afford the recommended diet were almost 3 times more likely to adhere to diet than those who could not afford it (AOR 2.9, 95% CI 1.0-8.3). Similarly, the participants having self-control on food were 4 times more likely to adhere on diet (AOR 4.1, 95% CI 1.2-14.1). Participants engaged in physical activities were 3.4 times more likely to adhere to diet (AOR 3.3, 95% CI 1.2-9.2). Participants who adhered to medication were 3.5 times more likely to adhere to diet (AOR 3.5, 95% CI 1.2-10.1).

## 4. Discussion

Our study shows that one-seventh of the participants have good adherence to diet. Among different factors analyzed for dietary adherence, namely, sociodemographic factors, disease-related factors, dietary factors, anthropometry, and medication adherence, we found that adherence to diet is more related with the variables, their self-control on food, their affordability on diet, their adherence on physical activity, and medication. Among the social factors, type of family is related to dietary adherence where single family is more likely to adhere on diet.

Our study shows only 15.7% had adherence to diet. A similar finding was seen in Kathmandu with the adherence rate of 14.29% [17], and in Dhaka, Bangladesh, adherence was 12% [18]. This similarity in the finding could be due to similar sociocultural environment. However, the adherence to dietary recommendation was 50% in Kolkata [19] and 84.6% in Delhi [20]. The reason for such contradictory finding could be the use of different tools to measure adherence. In this study, we measured adherence through Perceived Dietary Adherence Questionnaire. The study on Kolkata measured adherence based on the Indian guidelines, while in Delhi, the Summary of Diabetes Self-Care Activities tool was used.

There is a significant association between adherence to diet and type of family in our study where participants living in single family are 2.7 times likely to adhere to diet than the participants from the joint family. A similar finding was seen in the study done in Nepalgunj [14] as well as South India [21]. The similarity of the findings might be due to similar sample size, sampling techniques, and sociocultural environment. The possible reason for the nuclear family to be more adherent to diet in our context could be that the food varieties may sustain for longer time in nuclear family. Moreover, in the case of nuclear family, individual preference and dietary requirement could be addressed where it may not be possible in joint family. On bivariate analysis, our study shows that participants having formal education are more likely to adhere to diet than those having no formal education with a *p* value of 0.017. A similar finding was seen in bivariate analysis where illiterate was more noncompliant than literate [22]. This finding is supported by the study done in Bangladesh [18], Saudi [23], and Ethiopia [15]. This relation seems plausible as those getting formal education seek detailed information on their own regarding the disease and the importance of diet.

There is no relation between the type of treatment and adherence to diet. However, the study done in Saudi Arabia shows adherence to diet among those taking oral drugs than those taking both insulin and oral medications [23]. The possible reason for such contrasting result could be the low proportion of people taking both oral hypoglycemic drugs and insulin in this study (only 10.8%). So the proportion might not have been large enough to show the significant result. In our study, there was no association between comorbidity and adherence to diet. However, the study of Ethiopia showed that patients with the absence of comorbidity were more likely to adhere to diet than those with comorbidity [14]. Though the participants with comorbidity are higher in our study (54.9%), there is still a lack of routine health checkup in our context which might have altered the result. In various studies, the possible reason for inverse relation between comorbidity and dietary adherence was mentioned, which could be that the people with comorbidity have to follow dietary restrictions as per their disease condition and the complex dietary recommendation may be confusing.

The participants who could afford the recommended diet are almost 3 times more likely to adhere to diet than those who could not afford. Similar findings were shown in the study conducted in Ethiopia [15, 24, 25] where high cost of food was the reason for poor adherence. The relation seems plausible as those who can afford can have the choices of food for consumption. Similarly, in our study, participants having self-control on food were 4 times more likely to adhere on diet than those who do not have self-control. This finding is supported by the paper of Ganiyu et al. [26] and Adnan Iman [27]. This may be due to the use of self-reported data for measuring self-control habits rather than tools on all these studies.

The results of dietary adherence and physical activity adherence in our study show that participants are more compliant to moderate and vigorous physical activity (20.6%) than dietary recommendation (15.7%). With similar findings of dietary adherence (17.4%), physical activity adherence (10.4%) was observed in Iran [28]. In contrary to this finding, more people were dietary adherent (64.66%) and exercise adherent (45.33%) in Saudi Arabia [23]. This may be due to different cutoff values used for dietary adherence and physical activity adherence.

Our study shows that participants who were engaged in physical activities were 3.3 times more likely to adhere to diet than those who were not engaged. A similar finding was found in a study of Saudi Arabia [23]. A study conducted by Klinovszky et al. showed that adherence to diet showed a significant positive correlation with adherence to physical exercise which explained that the participants who attempted to integrate the diet prescribed for people with diabetes were more likely to follow the physical exercise regimen [29]. In our study, 60.8% of the participants were overweight and obese while 39.2% had normal BMI. A study in Addis Ababa showed that 46.4% of the participants were overweight and obese [24] while in Iran it was among 75.9% [28]. We found no relation between waist circumference and adherence to diet. However, the study of Raj et al. showed negative correlation between waist circumference and dietary adherence in the same study [30].

We found that almost half of the participants (52%) adhere to diabetes medication. A similar finding was reported on the study conducted in Kathmandu where medication adherence rate was 40.52% [22]. This may be because of the similar sociocultural environment of the participants. In contrast to the current study, high adherence rate (72.8%) of diabetes medication was observed in Iran [28]. Our study shows that those who adhere to medication were 3.5 times more likely to adhere to diet than those who do not adhere to medication. A contrary finding was observed by Klinovszky et al. where adherence to medication showed moderate-to-negative correlation with patients' adherence to diet. The findings suggested that the more patients adhere to taking antidiabetics regularly, the less motivated they feel to adhere to a proper diet [29]. Thus, this could reveal the need of appropriate counseling of patients for adherence to medication along with the adherence to dietary recommendations.

However, the study has some limitations which needed to be acknowledged. As we used a cross-sectional study design for the study, it cannot be used to infer the causality. Another limitation of this study was recall bias due to the retrospective nature of the data collection, possibly resulting in over- or underestimation of dietary practices. Although recall biases cannot be avoided, the researcher conducted all interviews by adequately probing questions for an attempt to gather exact information for revealing the real scenario of dietary adherence.

## 5. Conclusion

Our study shows that the adherence to dietary recommendation among people with type 2 diabetes is low. Type of family, affordability of recommended diet, self-control on food, physical activity, and medication adherence are significantly associated with adherence to dietary recommendations among people with type 2 diabetes. Health professionals and clinicians should be proactive in addressing these associated factors on giving dietary advice and adequate counseling on dietary recommendations. As research has not been done regarding the approaches used for counseling by the nutrition counselor, further study is recommended regarding the effectiveness of their counseling to assess the supply side of this problem.

## Figures and Tables

**Table 1:** Distribution of participants by their sociodemographic characteristics (*n* = 204).

Characteristics	Number	Percentage
Sex		
Female	116	56.9
Male	88	43.1

Age		
25-44	41	20.1
45-64	125	61.2
65 and above	38	18.7
*Mean* ± *SD* (in years)	53.03 ± 11.90	

Ethnicity		
Brahmin/Chhetri	109	53.4
Adibasi/Janajati	82	40.2
Dalit	13	6.4

Education		
Illiterate	67	32.8
Primary level	57	27.9
Secondary level	56	27.5
Higher secondary and above	24	11.8

Marital status		
Married	181	88.7
Single	23	11.3

Residence		
Urban	126	61.8
Rural	78	38.2

Religion		
Hindu	166	81.4
Buddhist	34	16.7
Christian	4	2.0

Occupation		
Non-income-generating work	116	56.86
Income-generating work	88	43.14

Type of family		
Single/nuclear family	113	55.4
Joint family	91	44.6

Family members		
<5	116	56.9
≥5	88	43.1

**Table 2:** Distribution of participants by disease-related, dietary factors, medication adherence, and anthropometric-related characteristics.

Disease-related conditions	Number	Percentage
Disease duration		
≤5 years	117	57.4
>5 years	87	42.6
*Mean* ± *SD* (in years)	6.17 ± 4.34	

Type of treatment		
Oral hypoglycemic drug	182	89.2
Oral hypoglycemic drug and insulin	22	10.8

Comorbidity		
Yes	112	54.9
No	92	45.1

Family history of diabetes		
No	152	74.5
Yes	52	25.5

Diet-related variables		
Sugar-sweetened beverage preference		
No preference	107	52.5
Preference	97	47.5

Affordability to healthy diet		
Affordable	105	51.5
Not affordable	99	48.5

Self-control on diet		
Self-control on food	133	65.2
No self-control on food	71	34.8

Place of food intake		
Home	198	97.1
Outside home	6	2.9

Anthropometric		
BMI		
18.5-24.9 (normal)	80	39.2
25-29.9 (overweight)	91	44.6
30-34.9 (obese I)	27	13.2
35-39.9 (obese II)	6	3

Waist circumference for male		
*Abnormal* > 85 *cm*	56	63.64
*Normal* ≤ 85 *cm*	32	36.36

Waist circumference for female		
*Abnormal* > 80 *cm*	77	66.38
*Normal* ≤ 80 *cm*	39	33.62

Waist-hip ratio for male		
>0.90	80	90.91
≤0.90	8	9.09

Waist-hip ratio for female		
>0.80	105	90.52
≤0.80	11	9.48

Medication adherence		
Adherent	106	52.0
Nonadherent	98	48.0

Dietary adherence		
Good adherence	32	15.7
Low adherence	172	84.3

**Table 3:** Factors associated with dietary adherence.

Study variables	Crude OR	Adjusted OR (95% CI)	*p* value
Education			
No formal education	Ref		
Formal education	2.6	1.4 (0.5-4.1)	0.454

Occupation			
Non-income-generating work	Ref		
Income-generating work	1.8	1.2 (0.4-3.2)	0.638

Type of family			
Joint family	Ref		
Single family	2.7	2.7 (1.0-7.4)	0.050^∗^

Sugar-sweetened beverage preference			
Preference	Ref		
No preference	2.6	2.0 (0.7-5.5)	0.136

Affordability to recommended diet			
Not affordable	Ref		
Affordable	5.1	2.9 (1.0-8.3)	0.042^∗^

Self-control on food			
No self-control on food	Ref		
Self-control on food	4.4	4.1 (1.2-14.1)	0.023^∗^

Physical activity			
No	Ref		
Yes	4.0	3.3 (1.2-9.2)	0.017^∗^

Medication adherence			
Medication nonadherent	Ref		
Medication adherent	4.9	3.5 (1.2-10.1)	0.021^∗^

^∗^Significant at *p* < 0.05. OR = odds ratio; Ref = reference category.

## Data Availability

The data used to support the findings of this study have been deposited in the public data repository platform “Figshare” and can be easily accessed using the link 10.6084/m9.figshare.20523111.
